# Comparative evaluation of rRNA depletion procedures for the improved analysis of bacterial biofilm and mixed pathogen culture transcriptomes

**DOI:** 10.1038/srep41114

**Published:** 2017-01-24

**Authors:** Olga E. Petrova, Fernando Garcia-Alcalde, Claudia Zampaloni, Karin Sauer

**Affiliations:** 1 Department of Biological Sciences, Binghamton Biofilm Research Center, Binghamton University, Binghamton, NY, USA; 2 Roche Pharma Research and Early Development, Immunology, Inflammation and Infectious Diseases, Roche Innovation Center Basel, F. Hoffmann-La Roche Ltd, Grenzacherstrasse 124, 4070 Basel, Switzerland

## Abstract

Global transcriptomic analysis via RNA-seq is often hampered by the high abundance of ribosomal (r)RNA in bacterial cells. To remove rRNA and enrich coding sequences, subtractive hybridization procedures have become the approach of choice prior to RNA-seq, with their efficiency varying in a manner dependent on sample type and composition. Yet, despite an increasing number of RNA-seq studies, comparative evaluation of bacterial rRNA depletion methods has remained limited. Moreover, no such study has utilized RNA derived from bacterial biofilms, which have potentially higher rRNA:mRNA ratios and higher rRNA carryover during RNA-seq analysis. Presently, we evaluated the efficiency of three subtractive hybridization-based kits in depleting rRNA from samples derived from biofilm, as well as planktonic cells of the opportunistic human pathogen *Pseudomonas aeruginosa*. Our results indicated different rRNA removal efficiency for the three procedures, with the Ribo-Zero kit yielding the highest degree of rRNA depletion, which translated into enhanced enrichment of non-rRNA transcripts and increased depth of RNA-seq coverage. The results indicated that, in addition to improving RNA-seq sensitivity, efficient rRNA removal enhanced detection of low abundance transcripts via qPCR. Finally, we demonstrate that the Ribo-Zero kit also exhibited the highest efficiency when *P. aeruginosa*/*Staphylococcus aureus* co-culture RNA samples were tested.

Studies of regulatory networks in bacteria often utilize analyses of gene expression. High-throughput *de novo* sequencing of transcriptomes or RNA-sequencing (RNA-seq) is a powerful tool for the global analysis of changes in transcript abundance. It is increasingly preferred over microarrays for its greater dynamic range, independence from probes, and alternative applications including discovery of new transcripts, mapping of transcription start sites, and sequencing of novel small RNAs. Despite reduced size, prokaryotic transcriptomes are nonetheless complex and provide unique analysis challenges. Bacterial transcriptomes contain protein-coding RNAs, transfer (t)RNAs, transfer messenger (tm)RNA, small regulatory (s)RNAs, and ribosomal (r)RNAs. The rRNA accounts for more than 85% of the prokaryotic cellular RNA content^1^, which can impede the analysis of mRNA transcripts, with ≥80% of library cDNAs mapping to rRNA in the absence of selection procedures^2^,^3^. In contrast to eukaryotic mRNAs, which can be selected using poly-A tails, polyadenylation of bacterial mRNAs is limited and indiscriminate and, thus, cannot be used for mRNA enrichment^4^. Therefore, approaches to address this issue have focused on removing the prokaryotic rRNAs prior to construction of cDNA libraries, with various methods developed including exonuclease treatment, polyadenylation^5^,^6^,^7^, electrophoretic size separation^8^, and subtractive hybridization capture of rRNA^9^,^10^.

The subtractive hybridization procedure, available as several commercial kits, has become the most common choice for rRNA depletion prior to prokaryotic RNA-seq analyses. Subtractive hybridization kits, such as the Ambion MICROBExpress™ Bacterial mRNA Enrichment Kit, which until recently has been considered to be one of the best and most widely used choices, rely on oligonucleotide probes to capture 16S and 23S rRNA. Such kits have been used on single-species cultures, multi-species communities, and environmental samples. The efficiency of these methods, however, has varied in a manner dependent on bacterial species and sample composition, with significant carryover of rRNA often observed following RNA-seq data analysis, with up to 50% of reads corresponding to rRNA^11^,^12^,^13^. Approaches to address such shortcomings have included repeated rounds of subtractive hybridization, combination of different methods, or the design and synthesis of custom capture oligonucleotides^11^,^12^.

An increasing number of studies have utilized RNA-seq to elucidate the regulatory processes behind growth and pathogenesis of *Pseudomonas aeruginosa*, an opportunistic human pathogen associated with infections of surgical sites, urinary tract, chronic and burn wounds, and lungs of cystic fibrosis patients, and one of the most frequent colonizers of medical devices. The aims of such studies have included identifying antisense and other small regulatory RNAs, determining the targets of regulatory proteins, assessing antibiotic resistance fitness costs, and analyzing the process of biofilm formation that represents a distinct mode of growth enabling the bacterium to colonize and persist within the infected host^14^,^15^,^16^,^17^,^18^,^19^. The efficiency of *P. aeruginosa* rRNA depletion by various methods, however, has not been evaluated. This is especially important considering that previous reports have demonstrated low yields of non-rRNA reads (5–30%) in cDNA libraries following depletion, with the problem being exacerbated in biofilm samples^18^. Despite a limited number of reports demonstrating better performance of the Ribo-Zero rRNA Removal Kit relative to the MICROBExpress kit in other bacterial species^13^,^20^, publication searches limited to 2016 for “*Pseudomonas aeruginosa”* or “biofilms” in combination with the respective kit names reveal similar numbers of projects utilizing the two procedures. Therefore, the present work was assessed the efficiency of three commercially available subtractive hybridization-based rRNA depletion kits: Illumina Ribo-Zero rRNA Removal Kit (Bacteria), Ambion MICROBExpress™ Bacterial mRNA Enrichment Kit and the Life Technologies RiboMinus Transcriptome Isolation Kit, Bacteria.

The MICROBExpress procedure employs capture oligonucleotides for 16S and 23S rRNA, which subsequently hybridize to oligonucleotides on derivatized magnetic beads to remove the rRNA. The RiboMinus procedure entails hybridization of 16S and 23S rRNA to rRNA sequence-specific 5′-biotin labeled oligonucleotide probes, which are then removed from the sample together with the bound rRNA using streptavidin-coated magnetic beads. Similarly, the Ribo-Zero kit relies on biotinylated rRNA capture probes, which, following hybridization to the target rRNA molecules, are captured by magnetic beads. In contrast to MICROBExpress and RiboMinus, the RiboZero kit also targets 5S rRNA. We provide evidence that, when tested using unmodified manufacturers’ protocols for the removal of rRNA from *P. aeruginosa* biofilm RNA samples, the Ribo-Zero kit outperformed the other two kits, reducing rRNA to less than 1% of all RNA-seq reads, and substantially improved the detection of low abundance transcripts during both RNA-seq and qPCR analyses. The Ribo-Zero kit also exhibited superior rRNA depletion efficiency when *P. aeruginosa* planktonic and *P. aeruginosa*/*Staphylococcus aureus* co-cultures samples were tested.

## Results

### Comparison of three commercially available rRNA depletion methods

**Figure 1:**
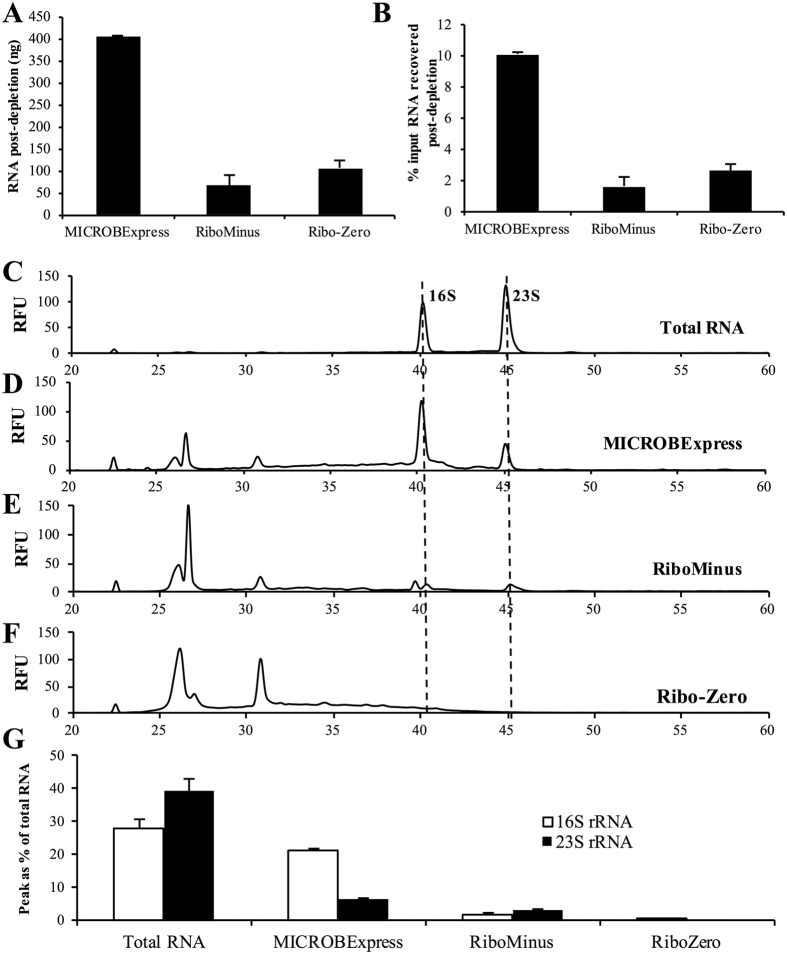
Quantitative and qualitative comparison of RNA recovered following rRNA depletion. A total of 4 μg of DNAse-treated RNA, isolated form 3-day-old *P. aeruginosa* PAO1 biofilms, was subjected to treatment with the Illumina Ribo-Zero rRNA Removal Kit (Bacteria), Ambion MICROBExpress™ Bacterial mRNA Enrichment Kit and the Life Technologies RiboMinus Transcriptome Isolation Kit, Bacteria. Following rRNA depletion and ethanol/acetate precipitation and resuspension in equal volumes of water, the RNA was assessed using Qubit fluorimetric quantitation with the Qubit RNA HS Assay Kit. Yields are reported as total RNA recovered (**A**) and as percentage of the input RNA (**B**). The RNA samples were also assessed using the Bioanalyzer RNA 6000 Pico kit. Representative electropherograms of (**C**) starting total RNA material and aliquots of the RNA samples that have been processed using the (**D**) MICROBExpress, (**E**) RiboMinus, or (**F**) Ribo-Zero kits are shown. Dashed lines indicate peaks corresponding to 16S and 23S RNA traces, which were detected in total RNA, MICROBExpress, and RiboMins samples. (**G**) The area of 16S and 23S rRNA peaks as percent of the total detected RNA was estimated, as determined using the 2100 Expert Software. RFU, relative fluorescence units. Experiments were repeated using three biological replicates.

In order to evaluate the efficiency of commercially available kits in the depletion of rRNA from *P. aeruginosa* biofilm samples, we have subjected 4 μg of DNAse-treated RNA isolated form 3-day-old PAO1 biofilms to treatment with the Illumina Ribo-Zero rRNA Removal Kit (Bacteria), Ambion MICROBExpress™ Bacterial mRNA Enrichment Kit and the Life Technologies RiboMinus Transcriptome Isolation Kit, Bacteria. In order to ensure valid comparison of the rRNA depletion methods, the procedures were performed on aliquots of the same input RNA sample, with the comparisons repeated using biological triplicates. Following rRNA depletion and ethanol/acetate precipitation, the resulting RNA was subjected to quantitative and qualitative analysis to determine yield and RNA species size distribution. Qubit fluorimetric analysis revealed the highest yield for the MICROBExpress-treated sample, with approximately 400 ng or 10% of the input RNA recovered (Fig. 1A,B). In contrast, less than 3% of input RNA was recovered in the RiboMinus and Ribo-Zero samples (65 ± 16 and 106 ± 25 ng of RNA, respectively).

We subsequently assessed whether the differences in RNA yields corresponded to differences in depletion of rRNA. The Agilent 2100 BioAnalyzer electrophoretic analysis revealed that the increased yield from the MICROBExpress procedure correlated with detectable presence of the 16S and 23S rRNA (Fig. 1C,D). The rRNA peaks, however, were significantly reduced in the MICROBExpress sample relative to the input total RNA (Fig. 1C,D). While the 16S and 23S rRNA peaks comprised close to 70% of the total detected RNA electropherogram area for the untreated RNA sample, these peaks represented ~30% of the MICROBExpress-treated RNA (Fig. 1G). In contrast, peaks corresponding to 16S and 23S rRNA detected in the RiboMinus sample comprised only ~5% of the total RNA electropherogram area (Fig. 1E,G). BioAnalyzer analysis of the the Ribo-Zero-treated samples revealed a peak in the range of 16S rRNA corresponding to 0.2% of the total RNA, with no 23S rRNA peak detected (Fig. 1F,G). These findings indicated that the MICROBExpress kit exhibits higher RNA yields at the cost of significant rRNA carryover, while the Ribo-Zero kit appears to deplete a significant portion of the rRNA present in the untreated RNA samples.

### qPCR assessment reveals differential levels of rRNA depletion

**Figure 2:**
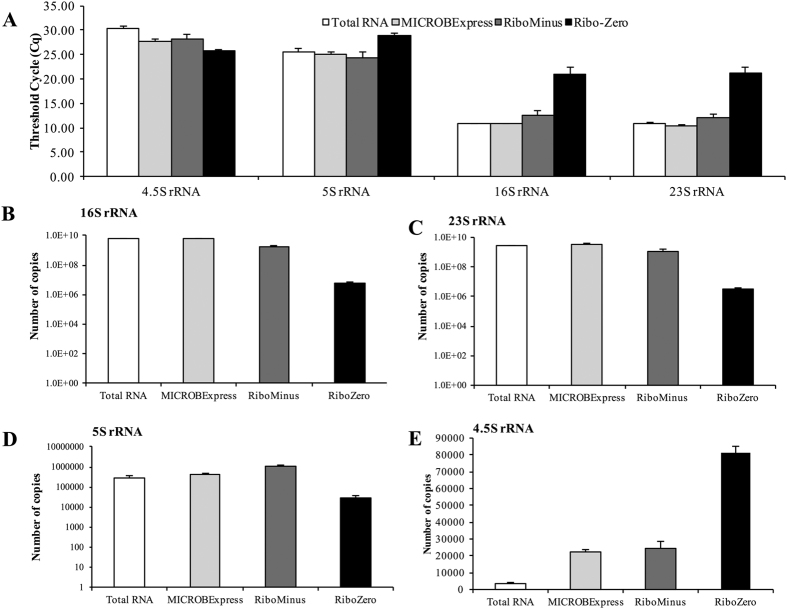
qPCR analysis of rRNA transcript abundance. (**A**) qPCR threshold cycle (C_q_) for the indicated rRNA transcripts. Copy numbers of (**B**) 16S, (**C**) 23 s, (**D**) 5S, and (**E**) 4.5S rRNA transcripts were calculated following the establishment of respective qPCR standard curves. cDNA synthesis for the qPCR reactions was performed using 10 ng of indicated RNA samples as input. Experiments were repeated using three biological replicates. Error bars indicate standard deviation.

Considering the observed differences in rRNA depletion efficiency, we next proceeded to verify these findings using quantitative (q)PCR. Following reverse transcriptase (RT)-PCR using the input untreated RNA and the three rRNA-depleted samples, the resulting cDNA was subjected to qPCR analysis using primers for *P. aeruginosa* 16S and 23S rRNA. Based on the BioAnalyzer data (Fig. 1), we anticipated the MICROBExpress sample to contain less rRNA transcripts than the total RNA, but significantly more than the other two rRNA-depleted samples, with the lowest rRNA transcript levels detected in the Ribo-Zero kit. As the qPCR threshold cycle (C_q_) corresponds to the amplification cycle when the product fluorescence (i.e. abundance) exceeds the background level, with lower C_q_ values corresponding to higher amounts of template, we accordingly expected the MICROBExpress sample to exhibit significantly higher C_q_ values than the input RNA, but lower C_q_ readings than the RiboMinus and Ribo-Zero samples, with Ribo-Zero demonstrating the highest C_q_ values. In agreement with this, the C_q_ values for 16S and 23S rRNA exceeded 20 for the Ribo-Zero samples, while products were detectable following 10–12 qPCR cycles in the MICROBExpress and RiboMinus samples (Fig. 2A). Surprisingly, the total RNA, MICROBExpress and RiboMinus samples exhibited similar C_q_ values in the range of 10–12 (Fig. 2A). Considering that equal amounts of RNA were used for cDNA synthesis, these results indicated significantly reduced abundance of rRNA in the Ribo-Zero, but not other rRNA-depleted samples. Accordingly, copy number calculations demonstrated similar 16S and 23S rRNA abundance in the total RNA and the samples treated with MICROBExpress and RiboMinus, but approximately 100- and 750-fold reductions in 16S and 23S rRNA copy numbers, respectively, in the Ribo-Zero sample relative to the untreated RNA control (Fig. 2B,C).

5S rRNA is an integral component of the large ribosomal subunit and is also present in the cell at relatively high levels. Thus, 5S rRNA can likewise be targeted for depletion prior to RNA-seq. However, only the Ribo-Zero kit specifically contains probes to remove the 5S rRNA species. In agreement with this, the qPCR analysis of the rRNA-depleted samples demonstrated significantly higher C_q_ values and lower copy numbers for 5S rRNA in the Ribo-Zero, but not the RiboMinus or the MICROBExpress samples relative to the input RNA (Fig. 2A,D).

In order to verify that the observed reductions in rRNA copy numbers were not due to compromised RNA quality, we also assessed the level of the 4.5S rRNA, which is not targeted by any of the three tested kits. Both the RiboMinus and MICROBExpress samples demonstrated significantly reduced C_q_ values for 4.5S rRNA relative to the total RNA sample, which corresponded to approximately 7-fold increases in 4.5S rRNA copy numbers (Fig. 2A,E). Moreover, 4.5S rRNA appeared to be approximately 27-fold more abundant in the Ribo-Zero samples relative to the total input RNA (Fig. 2A,E), indicating that the reduced levels of 5S, 16S, and 23S rRNA were not due to RNA degradation and suggesting that transcripts other than 5S, 16S, and 23S rRNA may be enriched in these samples. Together, these findings indicated that, when performed according to the manufacturers’ protocols, the Ribo-Zero rRNA depletion procedure is more efficient at reducing the amount of 16S and 23S rRNA. Moreover, these findings also confirmed that, out of the tested kits, only Ribo-Zero targets the 5S rRNA species, and that rRNA depletion may enrich and improve the detection of other RNAs as in the case of the 4.5S rRNA.

### Choice of rRNA depletion approach differentially affects RNA-seq analysis

**Figure 3:**
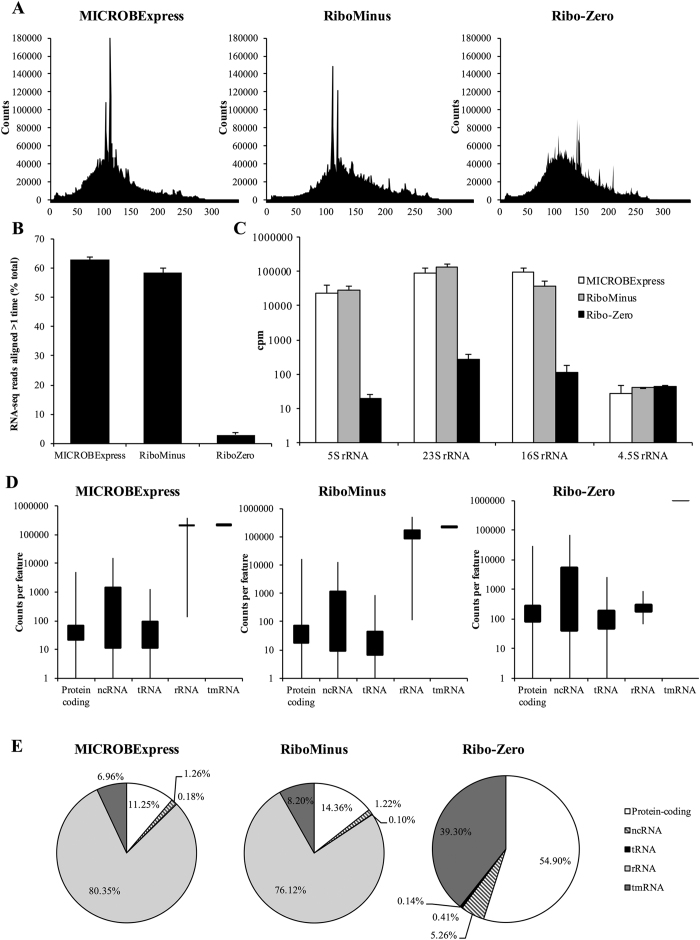
Ribo-Zero treatment reduces rRNA reads and increases non-rRNA reads during RNA-seq analysis. (**A**) Read length histogram from RNA-seq analysis of MICROBExpress-, RiboMinus-, and Ribo-Zero-treated samples performed on the Ion Torrent PGM system using the Ion PGM Template OT2 200 and Ion PGM Sequencing 200 v2 kits. Representative histogram from one RNA-seq run is shown. (**B**) Percentage of RNA-seq reads that aligned to more than one location on the *P. aeruginosa* PAO1 genome during bowtie-2 read mapping. (**C**) Counts per million (cpm) of detected reads matching rRNA-coding elements in the indicated RNA-sequencing samples, as determined by computing counts using Qualimap. Cpm, counts per million. Error bars indicate standard deviation. (**D**) Average number of counts detected per biotype for the indicated RNA-seq samples as determined using the NOISeq package software. (**E**) Counts detected per biotype as a percentage of total counts of the indicated RNA-seq runs. Data were derived from two independent RNA-seq experiments using biological replicates.

Given the differences observed in the profiles of the *P. aeruginosa* PAO1 3-day biofilm RNA samples following treatment with the three rRNA depletion methods, we subsequently assessed the effect of these kits on RNA-seq whole transcriptome analysis. Following cDNA library construction and sequencing on the Ion Torrent PGM system, diagrams of the size distribution of the sequenced reads, as reported by the PGM instrument, revealed that all three libraries had an average size distribution between 50 to 200 base pairs, but with markedly different patterns (Fig. 3A). While the Ribo-Zero-derived library exhibited a more even size distribution, those derived from the MICROBExpress- and RiboMinus-treated samples contained segments of sizes that were significantly overrepresented compared to the rest of the sample (Fig. 3A). In order to determine whether these were an overrepresentation of particular transcripts or a technical size-specific but sequence-independent enrichment of fragments, the RNA-seq data was subsequently mapped to the *P. aeruginosa* PAO1 genomic database.

Initial bowtie analysis revealed significantly higher percentages of reads mapping to more than one location in the genome for the MICROBExpress (62.9 ± 1.0%) and RiboMinus (58.3 ± 1.8%) samples than for the Ribo-Zero sample (2.9 ± 0.7%) (Fig. 3B). As four copies of each rRNA-encoding gene are present in the PAO1 genome and thus rRNA reads will map to more than one location in the genome, these findings, together with the size overrepresentation results (Fig. 3A), suggested the presence of increased numbers of rRNA tags in the MICROBExpress and RiboMinus samples. Subsequently, to assess potential differences in numbers of rRNA reads, Qualimap was used to compute and extract the counts for specific coding elements. The numbers of detected rRNA counts in the Ribo-Zero sample were reduced by 2–3 orders of magnitude (log_10_) compared to those detected in the MICROBExpress and RiboMinus samples (Fig. 3C). While the MICROBExpress and RiboMinus samples both produced rRNA counts in the range of 100,000 counts per million (cpm), the Ribo-Zero sample demonstrated rRNA detection at levels below 1000 cpm (Fig. 3C). The RNA-seq library sizes for all the samples were not significantly different, and all reported counts were normalized as counts per million. It is also of interest that tags for 4.5S rRNA, which is not targeted by any of the kits, did not significantly differ between the three samples (Fig. 3C). Together, these results confirmed the superior performance of the Ribo-Zero kit with respect to depletion of rRNA.

### RNA-seq detection of non-rRNA transcripts is enhanced by Ribo-Zero rRNA depletion

Given similar RNA-seq library sizes, a reduction in rRNA reads number would be expected to improve coverage of non-rRNA transcripts. To assess this, NOISeq was used to determine the counts distribution for various biotypes in the *P. aeruginosa* genome. Concurrently with lowering the rRNA reads number, the Ribo-Zero kit significantly increased the number of reads mapping to non-rRNA features including protein-coding transcripts, non-coding RNAs (ncRNAs), and tRNAs relative to the MICROBExpress and RiboMinus samples (Fig. 3D). Specifically, the mean cpm for protein-coding elements and ncRNAs were 5-fold higher in the Ribo-Zero samples relative to the other two. Moreover, compared to the MICROBExpress and RiboMinus samples, in which less than 15% of all reads mapped to protein-coding genes, more than 50% of the Ribo-Zero samples was composed of protein-coding transcripts (Fig. 3E). Similarly, percentage of reads mapping to ncRNAs, which are essential in the modulation of various processes including biofilm formation^21^, increased ~4-fold in the Ribo-Zero samples relative to the MICROBExpress and RiboMinus samples (Fig. 3E). In contrast, percentage of reads corresponding to rRNA decreased from 80% and 76% of total counts in the MICROBExpress and RiboMinus samples, respectively, to less than 0.15% of the counts in the Ribo-Zero samples (Fig. 3E).

Given the substantial reduction in rRNA counts, the protein-coding and ncRNA tags might be expected to account for more than 80% of the Ribo-Zero sample counts. However, together they only comprised 60% of the Ribo-Zero counts. The discrepancy was due to the transfer-messenger mRNA (tmRNA) SsrA, a bacterial RNA molecule with dual tRNA-like and mRNA-like properties, which was found to be the most abundant mRNA species in all three samples tested. Relative SsrA levels increased from 7–8% in the MICROBExpress or RiboMinus samples to close to 40% of all counts in the Ribo-Zero sample (Fig. 3D,E). Despite the abundance of SsrA counts, these findings strongly suggested that treatment of RNA samples with the Ribo-Zero kit substantially improves RNA-seq detection of non-rRNA transcripts relative to the MICROBExpress or RiboMinus modules.

### Ribo-Zero significantly improves RNA-seq coverage

**Figure 4:**
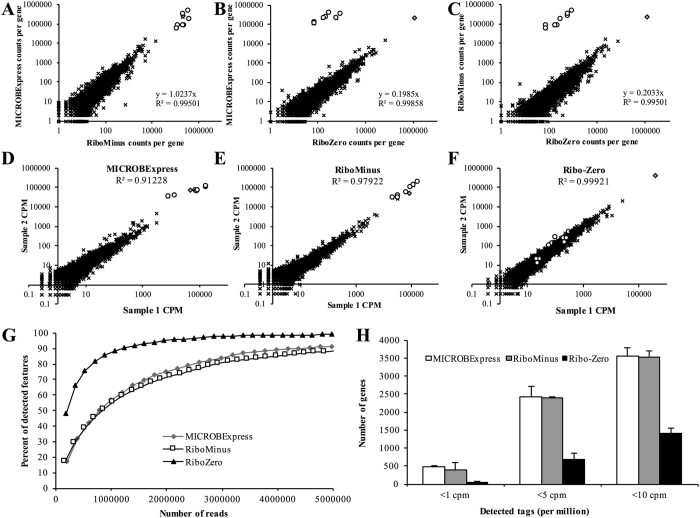
Ribo-Zero treatment improves RNA-sequencing depth. (**A**–**C**) Between-treatment comparison of tag distribution. In the scatter plots, each point indicates the counts per gene, as computed using Qualimap, in (**A**) MICROBExpress vs. RiboMinus, (**B**) MICROBExpress vs. Ribo-Zero, and (**C**) RiboMinus vs. Ribo-Zero samples. (**D**–**F**) Technical reproducibility of rRNA depletion methods. Each point in these correlation plots indicates the counts per gene in two replicates of (**D**) MICROBExpress, (**E**) RiboMinus, and (**F**) Ribo-Zero treatments performed on two biological replicates. In the scatter plots, rRNA-encoding regions are represented as white circles, the tmRNA *ssrA* is indicated as a grey diamond, and x’s represent all other transcripts. (**G**) Estimation of the sequencing depth of MICROBExpress, RiboMinus, Ribo-Zero RNA-seq samples as determined using the NOISeq software package. Data shown are based on two RNA-seq analysis using biological replicates. (**H**) Numbers of elements exhibiting low read counts (less than 1, 5, or 10 cpm) in the indicated RNA-seq samples. Error bars indicate standard deviation. cpm, counts per million.

Depth of sequencing coverage is a major consideration in transcriptome sequencing and performing quantitative analysis of transcript abundance. Thus, we next compared the sequencing depth of the data for the samples obtained using the different rRNA depletion methods. Global pairwise comparisons of the RNA-seq revealed that the MICROBExpress and RiboMinus samples demonstrated very similar distributions of tag mapping, with the slope approaching 1 (Fig. 4A), suggesting very similar levels of transcript detection in the two samples. The slopes were calculated using numbers of tags mapping to all elements with the exception of rRNA, in order to compare the coverage of non-rRNA transcripts. For the MICROBExpress to RiboMinus comparison, the rRNA data points (depicted as white circles in Fig. 4) clustered with the non-rRNA transcripts (black x’s in Fig. 4) and demonstrated the same 1:1 to relationship (Fig. 4A). In contrast, the average ratio of MICROBExpress to Ribo-Zero or RiboMinus to Ribo-Zero counts per non-rRNA gene/element was found to be approximately 0.2 (*R*^2^ > 0.995), suggesting a roughly five-fold enrichment of non-rRNA reads in the Ribo-Zero sample (Fig. 4B,C). For these MICROBExpress to Ribo-Zero or RiboMinus to Ribo-Zero comparisons, the rRNA data points clustered outside of those for non-rRNA transcripts, correlating with the significantly decreased numbers of rRNA reads in the Ribo-Zero samples (Fig. 4B,C).

Moreover, comparison of the RNA-seq counts of biological duplicates for each of the rRNA depletion kits revealed higher correlation of cDNA libraries produced following treatment with the Ribo-Zero kit (*R*^2^ = 0.99921) than the other two procedures (*R*^2^ = 0.91228 and 0.97922, for MICROBExpress and RiboMinus, respectively) when all transcripts were considered (Fig. 4D–F). When only non-rRNA targets were considered, the correlation for the MICROBExpress and RiboMinus replicates improved (*R*^2^ = 0.99458 and 0.99779, respectively), but was still below that observed for the Ribo-Zero replicates (*R*^2^ = 0.99921).

Furthermore, as a consequence of the increase in detected tags, it is not surprising that the Ribo-Zero sample demonstrated significantly improved sequencing depth relative to the MICROBExpress and RiboMinus samples (Fig. 4G). While signal saturation can be achieved with a total of ~3 million reads following Ribo-Zero rRNA depletion, samples treated with the RiboMinus or MICROBExpress kits will require in excess of 5 million reads to reach similar sequencing depth (Fig. 4G). Together, these findings indicated that the Ribo-Zero rRNA depletion results in both higher reproducibility and sequencing depth compared to the MICROBExpress and RiboMinus treatments.

### Ribo-Zero significantly improves detection of low abundance transcripts via RNA-seq

Low abundance transcripts are subject to higher variation due to background noise and are often eliminated from analysis. Increasing the sensitivity of RNA-seq experiments for such transcripts may facilitate the analysis of subtle, but important regulatory changes. Therefore, we next assessed the effect of the rRNA depletion methods on the incidence of low read counts. As anticipated, when elements with less than 1, 5, or 10 mapped cpm were considered, the Ribo-Zero samples exhibited significantly reduced incidence of low read counts relative to the other samples (Fig. 4H). For instance, while more than 400 genomic elements were detected with less than 1 cpm in the MICROBExpress and RiboMinus samples, only 53 elements on average had less than 1 mapped cpm in the Ribo-Zero sample (Fig. 4H). Similarly, when a threshold of 10 cpm was applied, in excess of 3500 genes were filtered out of the MICROBExpress and RiboMinus data sets, with ~1400 genes were eliminated in the Ribo-Zero sample (Fig. 4H).

**Figure 5:**
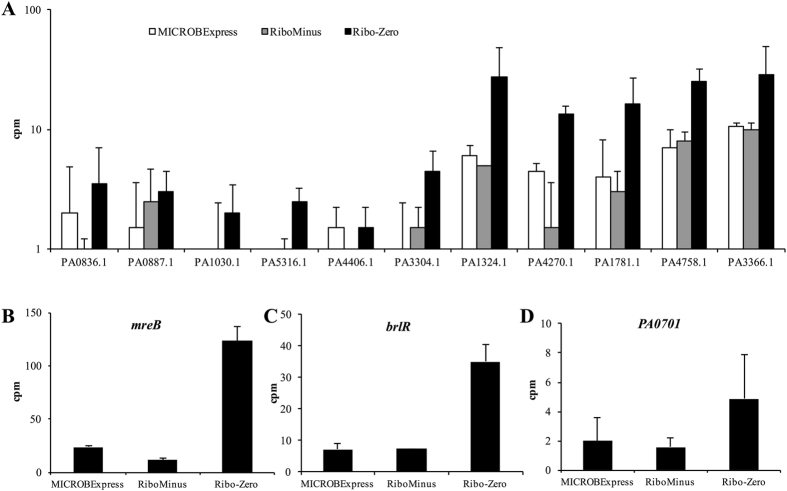
RNA-seq detection of low abundance transcripts is enhanced following Ribo-Zero treatment. Numbers of read counts per million detected for lower abundant (**A**) ncRNAs, (**B**) *mreB*, (**C**) *brlR*, and (**D**) PA0701 in the indicated RNA-seq samples. Data represent an average and are derived from two RNA-seq experiments using biological replicates. Error bars indicate standard deviation. cpm, counts per million.

We next compared the detection of low abundance ncRNAs, as well as of three protein-coding transcripts previously identified in our laboratory as being of low abundance during qPCR analysis. Specifically, we focused on the housekeeper control *mreB*^22^,^23^, *brlR* encoding a biofilm resistance regulator^24^, the locus *PA0701* encoding a transcriptional regulator previously associated with planktonic rather than biofilm growth^25^, as well as 11 ncRNAs for which a maximum of 100 cpm were detected. As expected, the Ribo-Zero sample demonstrated enhanced detection of lower abundance ncRNAs relative to the MICROBExpress and RiboMinus samples, with transcripts for PA1030.1 and PA5316.1 repeatedly detected only in the Ribo-Zero samples (Fig. 5A). Similarly, cpm for *mreB, brlR*, and PA0701 were up to 10-fold increase in the Ribo-Zero data sets relative to the MICROBExpress and RiboMinus sets (Fig. 5B–D). These observations suggested that, relative to other methods, Ribo-Zero rRNA depletion can reduce the number of genes removed using low count filters and thus facilitate a broader transcriptomic analysis.

### Ribo-Zero rRNA depletion improves qPCR sensitivity

We next proceeded to verify the RNA-seq findings using qPCR and to assess the impact of various rRNA depletion methods on qPCR efficiency. Specifically, we used qPCR to compare the levels of the lower abundance transcripts *mreB, brlR*, and *PA0701*, whose RNA-seq detection was improved via Ribo-Zero rRNA depletion (Fig. 5B–D). In contrast to the standard 500 ng–2 μg of input RNA, presently 10 ng of total RNA or MICROBExpress-, RiboMinus- or Ribo-Zero-treated RNA was used as a template for cDNA synthesis, given the low yield following rRNA depletion.

**Figure 6:**
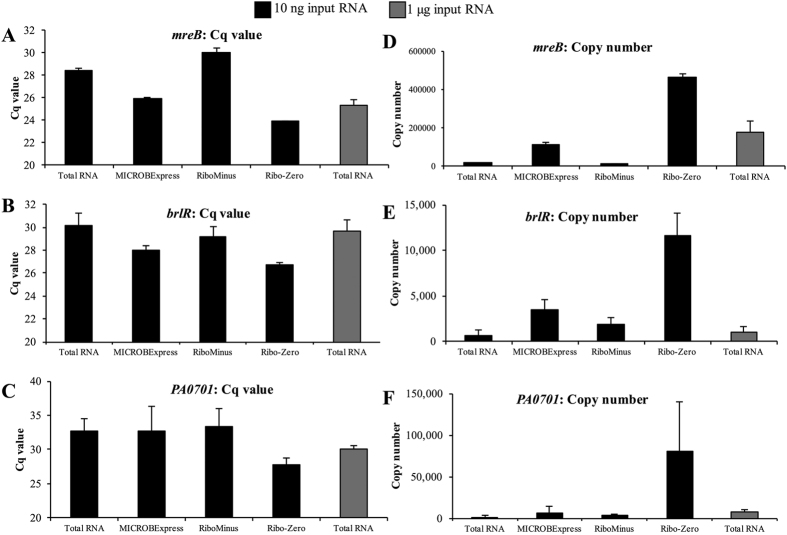
Ribo-Zero rRNA depletion improves transcript detection by qPCR. (**A**–**C**) qPCR threshold cycle (C_q_) for the indicated rRNA transcripts. (**D**–**F**) Copy numbers of *mreB, brlR*, and PA0701 transcripts were calculated following the establishment of respective qPCR standard curves. cDNA synthesis for the qPCR reactions was performed using 10 ng of total RNA rRNA-depleted RNA processed using the indicated kits. qPCR was also performed on cDNA generated from 1 μg of total RNA, with this sample used a control representing standard qPCR conditions. Experiments were repeated using three biological replicates. Error bars indicate standard deviation.

When the abundance of the housekeeper *mreB* transcript was tested, similar C_q_ values and transcript copy numbers were observed for the total RNA and RiboMinus samples (18,288 ± 2,882 and 11,790 ± 4,104; Fig. 6A,D). The MICROBExpress sample demonstrated ~5-fold increase in *mreB* transcript abundance, with 112,451 ± 11,898 copies detected on average. The largest increase was observed in the Ribo-Zero sample, with the detection of close to 500,000 *mreB* copies (Fig. 6A,D). Similar patterns were observed for *brlR* and *PA0701* (Fig. 6). These findings confirmed the observations obtained via RNA-seq and suggested that rRNA depletion using the Ribo-Zero procedure may be used to improve qPCR detection of lower abundance transcripts. Further evidence for this was obtained when a higher amount of untreated RNA was used for cDNA synthesis. Interestingly, the cDNA derived from 10 ng of the Ribo-Zero-treated sample exhibited significantly higher levels of *mreB, brlR*, and *PA0701* detection, relative to cDNA synthesized from not only 10 ng of total RNA, but also from 1 μg of total RNA (Fig. 6).

### Ribo-Zero outperforms the MICROBExpress kit in the depletion of rRNA from planktonic samples of Gram-negative and Gram-positive pathogenic bacteria

**Figure 7:**
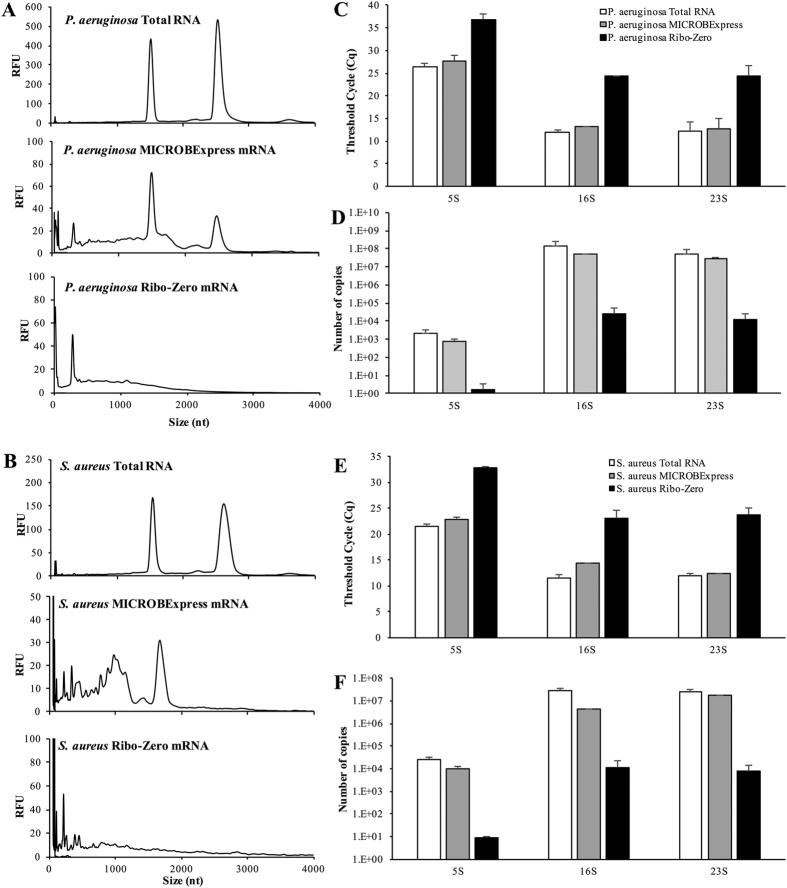
rRNA depletion treatments of RNA derived from planktonic *P. aeruginosa* PAO1 and *Staphylococcus aureus* ATCC6538 cells. A total of 2 μg of DNAse-treated RNA, isolated from exponential phase *P. aeruginosa* PAO1 or *Staphylococcus aureus* ATCC6538 cells, was subjected to treatment with the Illumina Ribo-Zero rRNA Removal Kit (Bacteria) or Ambion MICROBExpress™ Bacterial mRNA Enrichment Kit. Following rRNA depletion and ethanol/acetate precipitation and resuspension in equal volumes of water, the RNA was assessed using the Bioanalyzer RNA 6000 Pico kit. Representative electropherograms of starting total RNA material and aliquots of the RNA samples that have been processed using the MICROBExpress or Ribo-Zero kits are shown for the *P. aeruginosa* (**A**) and *S. aureus* (**B**) samples. The samples were subsequently subjected to qPCR analysis of rRNA transcript abundance, with qPCR threshold cycle (Cq) for the indicated rRNA transcripts shown for *P. aeruginosa* (**C**) and *S. aureus* (**E**). Copy numbers of *P. aeruginosa* (**D**) and *S. aureus* (**F**) 16S, 23 s, and 5S rRNA transcripts were calculated using respective qPCR standard curves. cDNA synthesis for the qPCR reactions was performed using 10 ng of indicated RNA samples as input. Experiments were repeated using two biological replicates. Error bars indicate standard deviation.

Having established the superior performance of the Ribo-Zero kit in the processing of *P. aeruginosa* biofilm RNA, we next asked whether these results are specific to *P. aeruginosa* biofilm samples, or whether similar results will be observed when planktonic samples of *P. aeruginosa*. Therefore, we compared the efficiency of the MICROBExpress and Ribo-Zero kits in the removal of rRNA from RNA samples derived from *P. aeruginosa* planktonic cells grown to the exponential stage. Only these two kits were subjected to subsequent analyses, as they are preferentially used over the RiboMinus kit. Similar to the results obtained for biofilm samples, Ribo-Zero outperformed the MICROBExpress kit, with Ribo-Zero-treated RNA from *P. aeruginosa* planktonic cells containing substantially less rRNA as revealed by Bioanalyzer (Fig. 7A,C,D). Moreover, based on qPCR analysis, the Ribo-Zero-treated sample contained >100-fold less copies of 5S rRNA and >1000-fold less of 16S and 23S rRNA than the sample processed with MICROBExpress (Fig. 7D).

In order to assess the range of their applicability, we also performed the comparative evaluation of the Ribo-Zero and MIROBExpress kits using RNA derived from the Gram-positive pathogen *S. aureus*, for which such an assessment has not been previously performed. Similar to the results obtained for the Gram-negative *P. aeruginosa* RNA samples, treatment of *S. aureus* RNA with the Ribo-Zero kit resulted in significantly reduced rRNA abundance relative to treatment with the MICROBExpress kit (Fig. 7B,E,F). Specifically, as revealed via qPCR analysis, the Ribo-Zero *S. aureus* mRNA samples contained >1000-less 5S, 16S and 23S rRNA relative to the samples processed with the MICROBExpress kit (Fig. 7E,F). Taken together, our findings not only indicated that the efficiency of the Ribo-Zero kit in depleting rRNA is independent of the mode of growth but also outperforms its competitors in the depletion of rRNA from both Gram-positive and Gram-negative bacterial samples.

### rRNA depletion efficiency does not differ for single- and dual-species samples

**Figure 8:**
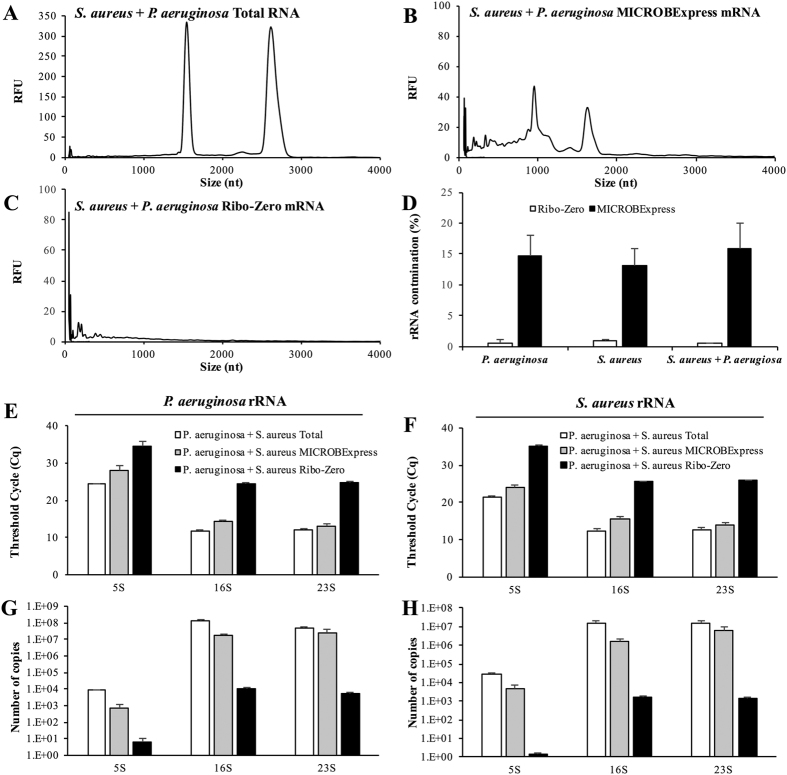
Depletion of rRNA from RNA samples isolated from dual species *P. aeruginosa*/*S. aureus* cultures. A total of 2 μg of DNAse-treated RNA, isolated form a dual species *P. aeruginosa* PAO1 and *S. aureus* ATCC6538 culture, was subjected to treatment with the Illumina Ribo-Zero rRNA Removal Kit (Bacteria) or Ambion MICROBExpress™ Bacterial mRNA Enrichment Kit. Following rRNA depletion and ethanol/acetate precipitation and resuspension in equal volumes of water, the RNA was assessed using the Bioanalyzer RNA 6000 Pico kit. Representative electropherograms of (**A**) starting total RNA material and aliquots of the RNA samples that have been processed using the (**B**) MICROBExpress or (**C**) Ribo-Zero kits are shown. (**D**) Percent of rRNA contamination as reported by the Agilent Bioanalyzer 2100 Expert Software in RNA samples from single and dual species cultures of *P. aeruginosa* and/or *S. aureus* treated with the Ribo-Zero or MICROBExpress kits. Untreated input RNA (Total) or RNA processed via Ribo-Zero or MICROBExpress treatment was subjected to qPCR analysis of *P. aeruginosa* (**E**,**G**) or *S. aureus* (**F**,**H**) rRNA transcript abundance. (**E**,**F**) qPCR threshold cycle (Cq) for the indicated rRNA transcripts. (**G**,**H**) Copy numbers of *P. aeruginosa* or *S. aureus* 16S, 23 s, and 5S rRNA transcripts were calculated using respective qPCR standard curves. cDNA synthesis for the qPCR reactions was performed using 10 ng of indicated RNA samples as input. Experiments were repeated using two biological replicates. Error bars indicate standard deviation.

Considering that *P. aeruginosa* and *S. aureus* are often found together in a clinically relevant polymicobial interaction that has been associated with infections of chronic wounds and the lungs of cystic fibrosis patients, we next assessed the efficiency of both rRNA depletion kits by processing RNA samples derived from a dual-species culture composed of these two pathogens. Following RNA isolation from the *P. aeruginosa* and *S. aureus* co-culture, the samples were subjected to rRNA depletion using the MICROBExpress or Ribo-Zero kits. Bioanalyzer electropherogram traces revealed that, while rRNA was reduced upon treatment with the MICROBExpress kit, little to no traces of rRNA were detectable upon treatment with RiboZero (Fig. 8A–C). Interestingly, the percentages of rRNA contamination, as reported by the Agilent Bioanalyzer 2100 Expert Software, were similar in RNA samples from single and dual species cultures of *P. aeruginosa* and/or *S. aureus* (Fig. 8D). Specifically, regardless of culture type, the estimated rRNA carryover for the Ribo-Zero samples was <1%, while the rRNA contamination in the MICROBExpress samples was estimated to be ~15%.

To further assess rRNA depletion from dual species RNA samples, qPCR was used by probing for the abundance of *P. aeruginosa* and *S. aureus* rRNA transcripts. Interestingly, the patterns of rRNA abundance for both *P. aeruginosa* and *S. aureus* were similar to those observed during the comparative evaluation using single-species cultures (Figs 7 and 8). Specifically, >1000-fold less *P. aeruginosa* and *S. aureus* 5S, 16S, and 23S rRNA was detected via qPCR in Ribo-Zero-treated samples than in untreated input samples or those processed with the MICROBExpress kit (Fig. 8E–H), indicating that the RiboZero kit is as efficient in depleting rRNA from samples obtained from single or dual species.

## Discussion

Despite their popularity, the efficiency of subtractive hybridization rRNA depletion kits has been variable and has often relied on user modifications, including custom rRNA probes and repeated rounds of the subtractive hybridization procedure. The present study represents the first comparative evaluation of the efficiency of rRNA depletion from *P. aeruginosa* and *S. aureus* single- and dual-culture RNA or from bacterial biofilm RNA samples. Our findings demonstrated that, for *P. aeruginosa* biofilm RNA samples, the Ribo-Zero rRNA removal procedure exhibited superior technical reproducibility and rRNA depletion efficiency and significantly improved RNA-seq sequencing depth relative to other commercially available subtractive hybridization kits. Moreover, the findings suggested that efficient rRNA depletion has applications beyond the scope of RNA-seq analysis, substantially enhancing the detection of low abundance transcripts by qPCR. Finally, the data demonstrated the superior efficiency of the Ribo-Zero kit in the depletion of rRNA not only from biofilm, but also from planktonic samples, as well as from single- and dual-species cultures of Gram-negative and Gram-positive pathogens.

The results of the present study caution against relying on nanofluidic electrophoretic analysis alone for the assessment of rRNA depletion. The BioAnalyzer electropherogram results suggested that treatment with either the MICROBExpress or the RiboMinus kit substantially reduces 16S and 23S rRNA abundance, with the RiboMinus procedure appearing to provide significanlty higher levels of rRNA deletion (Fig. 1). However, analysis of the rRNA-depleted samples by RNA-seq and qPCR demonstrated similar levels of rRNA detected following MICROBExpress and RiboMinus treatment (Figs 2 and 3). Given the differences in the electropherograms, these surprising results may possibly be attributed to a certain degree of RNA degradation during the RiboMinus procedure and potential carryover of degraded rRNA. Alternatively, despite significant reductions in abundance, 16S and 23S rRNA remain some of the highest abundance RNAs in the MICROBExpress and RiboMinus samples at levels approaching the higher limits of the dynamic ranges of detection (approaching saturation) of the RNA-seq and qPCR methods. Additional evidence for this comes from the observation that levels of 16S and 23S rRNA did not appear to be reduced in MICROBExpress and RiboMinus samples relative to total RNA according to qPCR, yet abundance of other transcripts was somewhat increased in these samples, indicating that non-rRNA enrichment did indeed occur (Figs 2 and 7).

Our results further suggested that, when compared to the MICROBExpress and RiboMinus procedures, the Ribo-Zero kit exhibits superior efficiency in the removal of 16S and 23S *P. aeruginosa* rRNA as revealed by BioAnalyzer, RNA-seq, and qPCR analyses. While in excess of 75% of all RNA-seq reads mapped to rRNA sequences in the MICROBExpress and RiboMinus samples, less than 0.5% of the reads in the Ribo-Zero sample corresponded to rRNAs (Fig. 3E). An additional advantage afforded by the Ribo-Zero procedure is the depletion of the *P. aeruginosa* 5S rRNA, as it is the only one of the three kits that contains oligonucleotide probes for these rRNA molecules. Our findings are in agreement with previous reports supporting the efficiency of Ribo-Zero rRNA depletion for other bacterial species. For instance, only the Ribo-Zero kit was capable of successfully depleting rRNA to below 1% in *Salmonella enterica* serovar Typhimurium strain SL1344 samples, with rRNA accounting for more than 90% of RNA-seq reads in MICROBExpress and RiboMinus samples^20^. When He and colleagues assessed MICROBExpress subtractive hybridization and an exonuclease treatment alone or in combination for rRNA depletion from samples derived from two synthetic polymicrobial communities, they found that even a combination of the two methods or repeated rounds of subtractive hybridization were capable of increasing non-rRNA reads to no more than 25%^11^. Similarly, MICROBExpress treatment failed to effectively remove rRNA from *Prochlorococcus marinus, Rhodobacter sphaeroides*, and *Escherichia coli*, samples^13^. In contrast, Ribo-Zero was found to remove the majority of rRNA from RNA of pure cultures of these bacteria, as well as from human stool samples^13^. Similarly, our findings indicated that Ribo-Zero efficiently removes rRNA (reducing rRNA abundance more than 1000-fold) not only from pure culture samples of the Gram-negative *P. aeruginosa* or the Gram-positive *S. aureus*, but also from a sample derived from a co-culture of these two pathogens.

Our results indicated that when *P. aeruginosa* biofilm RNA was subjected to subtractive hybridization using the Ribo-Zero kit, more than 99% of the RNA-seq reads mapped to non-rRNA sequences, with less than 0.1% and 0.5% tags matching rRNA and tRNA respectively (Fig. 3E). However, upon further biotype analysis, ncRNAs corresponded to 5% of reads and protein-coding tags were found to account for only 55% of all reads, with tmRNA SsrA corresponding to close to 40% of all reads (Fig. 3E). SsrA, possessing dual tRNA-like and messenger RNA-like properties, forms a ribonucleoprotein complex together with Small Protein B, Elongation Factor Tu, and ribosomal protein S1 and participates in *trans*-translation, facilitating recycling of stalled ribosomes and degradation of aberrant messenger RNA. Given the important role of SsrA, especially in survival under stressful conditions, it is not surprising that the tmRNA is present at high levels in the cell (Figs 3D,E and 4). Thus, these findings suggest that SsrA depletion may be an additional approach to improve RNA-seq coverage.

Ribo-Zero rRNA depletion afforded a significant improvement in sequencing depth relative to the other kits, with 3 million reads being sufficient for saturation coverage of the *P. aeruginosa* transcriptome (Fig. 4). Such improvement can ensure adequate transcriptome coverage for RNA-seq analyses performed on lower output systems such as the Ion Torrent PGM or the Illumina MiniSeq and MiSeq instruments. In addition, it can also facilitate increased multiplexing for multi-sample analyses on higher end systems such as the Ion Torrent Ion S5 or the Illumina NextSeq or HiSeq. The Ribo-Zero-facilitated enhanced sequencing depth translated into notably better coverage of lower abundance transcripts and a significant reduction in low count reads targeted by quality control filters (Figs 4 and 5). This improved detection of low abundance transcripts was not unique to RNA-seq, as similar results were obtained via qPCR. cDNA produced following Ribo-Zero treatment demonstrated enhanced amplification of *brlR, mreB*, and PA0701 relative to cDNA derived from equal amounts of MICROBExpress- or RiboMinus-treated RNA, as well as from an equal amount of untreated total RNA (Fig. 6). Moreover, similar results were observed even when 100-fold more untreated total RNA was used for cDNA synthesis: cDNA synthesized from 10 ng of Ribo-Zero-treated RNA contained higher levels of the respective non-rRNA transcripts than cDNA derived from 1 μg of untreated RNA (Fig. 6).

While rRNA depletion is essential for efficient RNA-seq analysis of gene expression patterns, some experiments may need to consider changes in rRNA transcript abundance. Previous findings have demonstrated that expression of rRNA-encoding genes is dependent on rate or mode of growth and changes in rRNA levels may be an important part of cellular regulatory responses^26^,^27^, with recent evidence suggesting that *P. aeruginosa* biofilm-specific mode of growth may be associated with differences in rRNA abundance^18^. Thus, analysis of such cellular responses may require alternative methods to supplement RNA-seq transcriptomic analysis to ensure that the importance of growth-associated changes in rRNA abundance is not overlooked.

## Materials and Methods

### Bacterial strains, media, and culture conditions

*P. aeruginosa* strain PAO1 and *S. aureus* strain ATCC6538 were used as indicated. All planktonic cultures were grown in Lennox Broth (LB, BD Biosciences) in flasks at 220 rpm. Biofilms were grown in 20-fold diluted LB medium using a continuous flow tube reactor system (1 m long size 13 silicone tubing, Masterflex, Cole Parmer, Inc.) with an inner surface area of 25 cm^2^ at a flow rate of 0.1 ml/min^28^,^29^. Following 3 days of growth, the biofilms were harvested by pinching the tubing, which resulted in the biofilm biomass being extruded from the inner surface of the tube reactor.

### RNA isolation

Samples of biofilm cells were collected as described above into 3 mL of RNAprotect Bacteria Reagent (Qiagen). Following 10-minute room temperature incubation, mRNA isolation (RNeasy Mini Kit, Qiagen) was carried out using approximately 3 × 10^8^ cells according to the manufacturer’s protocol, with the following modifications. For biofilm experiments, *P. aeruginosa* cells were treated with 400 μg/mL lysozyme in TE for 5 min at room temperature prior to RNeasy RNA isolation. For planktonic and co-culture experiments, cells were treated with 400 μg/mL lysozyme and 100 μg/mL lysostaphin in TE for 30 min at 37 °C prior to RNeasy RNA isolation. The RNeasy elution step was also modified to use 75 uL of 1X TE (10 mM Tris-HCl 1 mM EDTA) buffer to ensure downstream stability of the RNA during the RNA-seq library preparation. The RNA was subsequently subjected to DNase treatment (TURBO DNA-*free* DNase Treatment, Ambion) according to the manufacturer’s protocol. RNA quantity and quality were assessed as described below.

### RNA and DNA quantity and quality assessment

Where indicated, RNA or DNA was quantified using Qubit RNA HS Assay Kit and Qubit dsDNA HS Assay Kit (Life Technologies), respectively, on the Qubit 2.0 Fluorometer (Life Technologies). In addition, where indicated, fragment size distribution of RNA or DNA samples was assessed on the Agilent 2100 BioAnalyzer using the Agilent RNA 6000 Pico Kit or Agilent High Sensitivity DNA Kit (Agilent Technologies), respectively. The BioAnalyzer was also used to assess the quality of DNAse-treated total RNA, with only RNA samples exhibiting an RNA integrity number (RIN) >9 selected for downstream processing^30^.

### Ribosomal RNA (rRNA) depletion

A total of 4 ug RNA was subjected to rRNA depletion using the Illumina Ribo-Zero rRNA Removal Kit (Bacteria), Ambion MICROBExpress™ Bacterial mRNA Enrichment Kit and the Life Technologies RiboMinus Transcriptome Isolation Kit, Bacteria, according to the manufacturers’ protocols. RNA quantity and quality post-depletion was assessed using the Agilent 2100 Bioanalyzer instrument as described above. In order to ensure valid comparison of the rRNA depletion methods, the procedures were performed on aliquots of the same input RNA sample, with the comparisons repeated using biological triplicates.

### RNA-Seq library construction and sequencing

Whole transcriptome RNA-Seq was carried out using the ION Torrent Personal Genome System (Life Technologies). Following rRNA depletion and BioAnalyzer quality assessment performed as discussed above, the cDNA libraries was constructed using the Ion Total RNA-Seq kit v2 (Life technologies) according to the manufacturer’s protocol. Following BioAnalyzer size distribution assessment and Qubit quantitation, the cDNA libraries were used to prepare sequencing templates using the Ion PGM Template OT2 200 kit on the Ion OneTouch™2 instrument (Life Technologies), followed by Ion Sphere™ Quality Control Kit assessment and template-positive Ion Sphere™ Particle (ISP) enrichment using the Ion OneTouch™ ES instrument. Sequencing was carried out using Ion Sequencing 200 Kit v2 and the Ion 318 Chip on the Ion Torrent PGM sequencing platform (Life Technologies). For each rRNA depletion method, RNA-sequencing was performed in duplicate using distinct biological replicates. The raw sequencing data are available in the NCBI short read archive (http://www.ncbi.nlm.nih.gov/Traces/sra/sra.cgi) with accession number SRP072237 under BioProject PRJNA316121.

### RNA-Seq data analysis

FastQC (Babraham Bioinformatics, [http://www.bioinformatics.bbsrc.ac.uk/projects/fastqc/]) was used to generate statistics and evaluate the quality of the generated RNA-seq reads. Bowtie 2^31^ with default parameters was applied to map the RNA-seq reads to the *P. aeruginosa* PAO1 reference genome^32^ and subsequently converted to BAM format for downstream analysis using SAMtools^33^. Qualimap was then used to generate alignment statistics and to compute the number of counts per genomics element^34^. In addition, biotype distribution and depth of coverage analyses were performed using the R package NOISeq^35^.

### Quantitative real-time reverse-transcription PCR (qPCR)

cDNA synthesis was performed using the iScript™ cDNA Synthesis Kit (BioRad), with 10 ng or 1 μg of total RNA or 10 ng of rRNA-depleted RNA (processed with MICROBExpress, RiboMinus, or Ribo-Zero kit) used as the template. Subsequently, qPCR for indicated transcripts was performed using the BioRad CFX Connect Real-Time PCR Detection System and SsoAdvanced™ SYBR^®^ Green Supermix (BioRad) with oligonucleotides listed in Table 1. In order to establish standard curves for rRNA, *mreB, brlR*, and PA0701 transcript copy numbers versus C_q_ values, as calculated using the CFX Manager Software (BioRad), qPCR was also performed using serial 10-fold dilutions of *P. aeruginosa* genomic DNA. Subsequently, the standard curves were used for calculations of template copy numbers in the indicated samples. Melting curve analyses were employed to verify specific single product amplification. The qRT-PCR analysis was performed using biological triplicates.

## Additional Information

**How to cite this article:** Petrova, O. E. *et al*. Comparative evaluation of rRNA depletion procedures for the improved analysis of bacterial biofilm and mixed pathogen culture transcriptomes. *Sci. Rep.* **7**, 41114; doi: 10.1038/srep41114 (2017).

**Publisher's note:** Springer Nature remains neutral with regard to jurisdictional claims in published maps and institutional affiliations.

## Figures and Tables

**Table 1:** Oligonucleotides used in this study.

Name	Sequence
16S_RT_for	GACTCCTACGGGAGGCAG
16S_RT_rev	GACTCCTACGGGAGGCAG
23SrRNAssoFor	GAGTAGGACGGAGCACGAG
23SrRNAssoRev	CTTGTACGCATACGGTTTCAG
5S_RT_for	GCTTGACGATCATAGAGCG
5S_RT_rev	CTTGACGATGACCTACTCTC
4.5S_RT_for	CGTCAACCTGGTCAGGTCC
4.5S_RT_rev	GCAGCGCTACCGATAAGAAC
PA0701f	CTATCGGAGTCCGCCGG
PA0701r	GTAGCCCCACAGCTCGC
mreB-RT-CFX-for	CTTCATCAACAAGGTCCACGA
mreB-RT-CFX-rev	GCTCTTCGATCAGGAACACC
brlR_RT_for	GCAACGACACCAGCACAC
brlR_RT_rev	GAAGCGTTCCCAGAGCTG
Saur_16S_RT_for	GTGAAAGACGGTCTTGCTGTC
Saur_16S_RT_rev	GGAAGATTCCCTACTGCTGC
Saur_23S_RT_for	GCACACCCGGAGAACTGAAAC
Saur_23S_RT_rev	GATGATTCGTCTAATGTCGTCC
Saur_5S_RT_for	CTGGTGACTATAGCAAGGAGG
Saur_5S_RT_rev	CCTGGCAACGTTCTACTCTAG
